# Incidence, diagnosis and management of tubal and nontubal ectopic pregnancies: a review

**DOI:** 10.1186/s40738-015-0008-z

**Published:** 2015-10-15

**Authors:** Danielle M. Panelli, Catherine H. Phillips, Paula C. Brady

**Affiliations:** 1 Department of Obstcpetrics and Gynecology, Brigham and Women’s Hospital, Harvard Medical School, 75 Francis St., Boston, MA 02115 USA; 2 Department of Radiology, Brigham and Women’s Hospital, Harvard Medical School, Boston, MA USA

**Keywords:** Ectopic pregnancy, Nontubal ectopic pregnancy, Heterotopic pregnancy

## Abstract

**Background:**

Ectopic pregnancy is a potentially life-threatening condition occurring in 1-2 % of all pregnancies. The most common ectopic implantation site is the fallopian tube, though 10 % of ectopic pregnancies implant in the cervix, ovary, myometrium, interstitial portion of the fallopian tube, abdominal cavity or within a cesarean section scar.

**Findings:**

Diagnosis involves a combination of clinical symptoms, serology, and ultrasound. Medical management is a safe and effective option in most clinically stable patients. Patients who have failed medical management, are ineligible, or present with ruptured ectopic pregnancy or heterotopic pregnancy are most often managed with excision by laparoscopy or, less commonly, laparotomy. Management of nontubal ectopic pregnancies may involve medical or surgical treatment, or a combination, as dictated by ectopic pregnancy location and the patient's clinical stability. Following tubal ectopic pregnancy, the rate of subsequent intrauterine pregnancy is high and independent of treatment modality.

**Conclusion:**

This review describes the incidence, risk factors, diagnosis, and management of tubal and non-tubal ectopic and heterotopic pregnancies, and reviews the existing data regarding recurrence and future fertility.

## Findings

An ectopic pregnancy (EP) refers to the implantation of an embryo outside of the uterus. Due to advances in laboratory testing, transvaginal ultrasound, chemotherapy and laparoscopy, the evaluation, diagnosis and management of EP has rapidly evolved. In parallel, maternal mortality has declined, from 3.5 of 10,000 pregnancies in 1970 to 2.6 of 10,000 in 1992 [1].

The most common EP location is in the fallopian tube, predominantly the ampullary region of the fallopian tube. Implantation outside the fallopian tube—in the cervix, ovary, myometrium, abdominal cavity, interstitial (i.e., intramuscular/proximal) portion of the fallopian tube or coincidentally with an intrauterine pregnancy—occurs in less than 10 % of EPs. Heterotopic pregnancy (HP) refers to the coexistence of an intrauterine pregnancy with an EP in any of these locations. ‘Cornual’ pregnancies are those implanted in a horn of an anomalous uterus (i.e., unicornuate, bicornuate, didelphys or septate uteri); these do not uniformly require intervention and will not be included in this review [2–4].

This review will describe the incidence, risk factors, diagnosis and management of women with tubal and nontubal EPs, as well as review the existing literature regarding their future fertility.

## Review

### Incidence

The overall rate of EP is 1–2 % in the general population, and 2–5 % among patients who have utilized assisted reproductive technology (ART) [5, 6]. Although the overall mortality has decreased over time, ruptured EPs still account for up to 6 % of all maternal deaths; a review of mortality in ART-associated EPs similarly reported a mortality rate of 31.9 deaths per 100,000 pregnancies [5, 7].

Nontubal EPs are pregnancies that implant at sites other than the fallopian tube. These pregnancies account for less than 10 % of all EPs, though their overall incidence has been increasing in recent years [5]. Furthermore, nontubal EPs contribute disproportionately to maternal morbidity and mortality in comparison to tubal EPs. Cervical EPs are estimated to occur in 1:2000 to 1:18,000 pregnancies [8]. The estimated incidence of cesarean scar EPs is 1:1800 to 1:2216 pregnancies, or 6 % of all EPs in women with at least one cesarean delivery [9, 10]. Interstitial EPs account for 4 % of EPs, though the associated morbidity is much higher, with mortality rates of 2.5 % or 7 times the mortality rate associated with other EP locations, largely due to hemorrhage [11, 12]. Pregnancies embedded within the myometrium (intramural EPs) account for an estimated 1 % of EPs [13]. Abdominal pregnancies account for 1.3 % of EPs [14]. These have been classified as primary or secondary; secondary abdominal EPs are theorized to result from extrusion from the fallopian tube and subsequent intraabdominal reimplantation [15]. Most common implantation sites are in the pouches posterior and anterior to the uterus and on the serosa of the uterus and adnexa; retroperitoneal, omental, bowel, hepatic and splenic implantations have also been reported [16].

Estimates of the incidence of heterotopic pregnancy (HP) vary by article and decade; the risk has been reported from 1:4000 to 1:30,000 women in the general population [5, 17]. The risk of HP following in vitro fertilization (IVF) has been estimated as high as 1:100 women [5, 17, 18]. HPs can include an EP in any of the previously described locations; a triplet HP that included tubal and cervical EPs has even been described [19]. The majority are tubal HPs; in a review of 80 cases of HP in the literature, 66 (72.5 %) were in the ampullary or interstitial portion of the fallopian tube, while 7 were cervical and 3 were implanted in the cesarean scar [17].

### Etiology of tubal ectopic pregnancy

The fallopian tube is a carefully controlled environment to facilitate oocyte transport, fertilization, and migration of the early embryo to the uterus for implantation [20] Most data suggest tubal EP stems from both abnormal embryo transport and an alteration in the tubal environment, which enables abnormal implantation to occur [21].

The transport of an oocyte and embryo through the tube relies on both smooth muscle contraction and ciliary beating, which are affected by several local factors—toxic, infectious, immunologic and hormonal. Smoking and infection have been shown to decrease cilia density, while ciliary beat frequency has been shown to be responsive to the changing hormonal milieu of the menstrual cycle [22–24]. Samples of fallopian tube epithelium incubated in estradiol (E2) and nitric oxide (NO) have been found to demonstrate increased ciliary motility, which may cause aberrant tubal transport [25, 26]. NO also affects smooth muscle contractility in the fallopian tube; expression of NO has been found to vary during the menstrual cycle, with possible implications for normal and ectopic implantations [27]. Finally, E2-mediated effects via estrogen receptors on gene regulation and expression—including pathways implicated in implantation and apoptosis—may be involved in aberrant tubal function and ectopic pregnancy, though more research is needed to clarify these pathways [28–30].

Inflammation in the fallopian tubes is also implicated in the establishment of EP, by inducing tubal dysfunction or damage that may lead to retention of an oocyte or embryo, and by promoting embryo implantation in the fallopian tube via inflammatory cytokines [31]. Following tubal damage by smoking or infection, upregulation of pro-inflammatory cytokines has been noted, promoting embryo receptivity, invasion and angiogenesis in the tube. For instance, interleukin 1 (IL-1), produced by tubal epithelial cells following *Chlamydia trachomatis *infection, is a vital signal for embryo implantation in the endometrium; IL-1 also recruits neutrophils downstream, leading to further tubal damage [32]. Macrophages and intraepithelial lymphocytes are also increased in women with EP, potentially affecting tubal function and predisposing to tubal EP [33–35].

### Clinical risk factors

Up to 50 % of women diagnosed with EPs have no identifiable risk factors; however, a number of risk factors have been associated with EP [5]. These include age, smoking, history of EP, tubal surgery or tubal damage, prior pelvic infection, DES exposure, IUD use and pregnancy conceived by assisted reproduction.

Age has been shown to be a risk factor for EP, with the highest incidence over the age of 35 in both spontaneous pregnancies and those conceived after assisted reproductive technologies [7, 36]. The explanation for this observation is unknown, however age is theorized to affect tubal function, including delay of oocyte transport [36, 37].

Prior EP is a strong risk factor for recurrent EP, with a recurrence rate of 5–25 %, or up to 10 times the risk in the general population [38–40]. Prior treatment for EP, whether medical or surgical, may result in pathologic changes in tubal motility, ciliary function and uterine contractions [41]. (For fertility outcomes after prior EP, please see “Recurrence and future fertility”).

Smoking is thought to increase the risk of EP by causing tubal dysfunction, including deciliation [22]. Tobacco may cause dysregulation of the paracrine signals needed for coordinated embryo transport and development [21]. In a retrospective review of 481 IVF cycles, the likelihood of a tubal EP was three times higher among smokers [41]. A dose-dependent relationship between smoking and EP has also been confirmed in spontaneous pregnancies [36].

A history of pelvic infection or pelvic inflammatory disease is associated with increased risk for subsequent EP. *Chlamydia trachomatis* in particular has been implicated as a risk factor for EP, with the risk increasing with each successive infection; ascending infection and resultant salpingitis is thought to lead to tubal dysfunction and aberrant implantation [5, 21, 42, 43]. Other infections potentially associated with pelvic inflammatory disease and tubal damage include *Neisseria gonorrhoeae, Mycoplasma* and schistosomiasis [44, 45].

Prior tubal surgeries, including but not limited to tubal reanastamosis, salpingostomy, tuboplasty and lysis of adhesions, are risk factors for EP. Similarly, any causes of pelvic adhesions, including endometriosis, appendicitis, or other pelvic surgeries, may distort the anatomy of the fallopian tube [46, 47]. The risk of EP after tubal reanastamosis, specifically, is estimated at 2–13 %, and is similar between abdominal and robotic approaches [6, 48].

After surgical sterilization, the failure rate across all sterilization methods is estimated at 18.5 per 1000, approximately one-third of which are EPs [49]. Women sterilized before 30 years of age are twice as likely to have subsequent EPs as those sterilized after age 30 years. Rates of EP vary by sterilization technique: After bipolar coagulation, 65 % of pregnancies are EP, while after unipolar or clip sterilization, approximately 15 % of pregnancies are EP [50]. The proportion of EP has been shown to be three times higher between 4 and 10 years after sterilization, as compared to the first 3 years [49].

Current IUD use dose not predispose to EP, though a higher proportion of pregnancies conceived with an IUD in place are ectopic as compared to the general population [43, 51]. Among pregnancies conceived with IUDs in place, half are ectopic with a levonogestrel device in place, compared to 1:16 with a copper IUD in place [52]. Overall, any contraceptive use decreases the risk of both intrauterine and ectopic pregnancy.

Assisted reproductive technologies constitute a risk factor for EP, as 2–5 % of pregnancies from assisted reproductive technologies are ectopic [7]. The three main factors contributing to this increased risk are the specific type of procedure, the reproductive health characteristics of the woman, and the estimated embryo implantation potential [21, 53]. A history of infertility, even in the absence of known tubal disease, is associated with EP, with the EP risk increasing with a longer duration of infertility [18, 43]. Tubal factor infertility specifically is associated with a two-fold risk of EP following IVF [52, 54, 55].

Several IVF cycle parameters may be associated with an increased risk of EP. Patients undergoing cycles triggered with gonadotropin releasing hormone (GnRH) agonists instead of recombinant hCG may be at higher risk of EP; in a review of 466 IVF cycles, GnRH agonist triggers were associated with a significantly higher EP rate (5.3 % versus 1.4 % following hCG triggers). This finding is theorized to be due to poor endometrial receptivity following GnRH agonist administration [56].

The number of embryos transferred may be correlated to the EP risk; in a review of 9480 ectopic pregnancies following IVF, the rate of EP following fresh cycles rose significantly from 1.7 % following single embryo transfer to 2.5 % following the transfer of 4 embryos [7]. Depth of transfer may also have an effect; a randomized prospective study of deep versus mid-fundal transfer reported an EP rate of 1.5 versus 0.4 % [57]. Day of embryo transfer has inconsistently been associated with risk of EP in prior studies. A series of 13,654 fresh cycles reported an EP rate of 2.1 % following day 3 embryo transfers, as compared to a rate of 1.6 % following day 5 embryo transfers, which did not reach statistical significance [58]. Conversely, a review of 1994 fresh transfers reported a significant difference in ectopic pregnancy rates between day 3 and day 5 transfers, at 2.4 and 1.7 %, respectively [59].

The transfer of fresh embryos is associated with a higher EP risk as compared to the transfer of thawed embryos; following 15,042 fresh cycles, the EP rate was 1.97 %, which was significantly higher than the 1.01 % EP rate following 12,752 cryopreservation cycles [55]. It is theorized that the controlled ovarian hyperstimulation and hyperestrogenic environment preceding a fresh embryo transfer negatively effects endometrial receptivity [60].

### Risk factors for nontubal EP

Overall, the risk factors for ovarian EPs, interstitial EPs, and tubal HPs are similar to those for tubal pregnancy. These include a history of a prior EP, pelvic infections and use of in vitro fertilization [61]. The transfer of four or more embryos during IVF is an additional risk factor for HP [18, 62]. IUD use may be a risk factor for nontubal EP, particularly for ovarian EP [63]. An additional risk factor for interstitial implantation includes prior ipsilateral salpingectomy, with interstitial ectopic pregnancies occurring up to 13 years after salpingectomy [4]. Risk factors for other specific types of nontubal EPs are outlined in the following subsections.

### Intramural EP

Risk factors for these rare EPs are theorized to include myometrial injury following uterine curettage, and prior myomectomy or cesarean section [13]. Assisted reproductive technologies have been used in approximately 20 % of case reports, and another 19 % of patients carried a diagnosis of adenomyosis.

### Cesarean section EP

Risk for cesarean scar implantation is not clearly correlated to the number of prior cesarean sections [64]. Risk for cesarean section scar implantation has not been correlated to single versus double layer closure of the hysterotomy at the time of cesarean section. Cesarean scar implantation may be more common following cesarean sections for elective indications, which is theorized to be due to impaired healing of an unlabored lower uterine segment [65].

### Cervical EP

A history of dilation and curettage (D&C) in a previous pregnancy has been associated with subsequent cervical EP; this risk factor is present in nearly 70 % of cases [66, 67] In-vitro fertilization has been proposed as a risk factor, but often coincides with D&C and other possible risk factors, so is difficult to isolate as an independent contributor to risk [68].

### Abdominal pregnancy

Risk factors for abdominal pregnancy are similar to those for tubal EPs, including pelvic inflammatory disease, use of assisted reproductive technologies and endometriosis [69]. Most abdominal pregnancies have been published in case reports; one details the occurrence of a twin pregnancy implanted in the broad ligament after IVF. Uterine perforation was suggested as a possible cause as the embryo transfer was performed using a stylet, which is more rigid than standard transfer catheters [70].

### Diagnosis

#### Serum beta-human chorionic gonadotropin (β-hCG)

The diagnosis of EP often begins with the preliminary diagnosis of pregnancy of unknown location (PUL). PUL is defined as a positive serum beta-human chorionic gonadotropin (β-hCG) in the absence of ultrasound findings indicative of intrauterine or extrauterine pregnancy. Approximately 30 % of patients with PUL will develop an ongoing intrauterine pregnancy (IUP), while the majority (50–70 %) will be diagnosed with failing pregnancies, either miscarriages or EPs [71].

In the stable patient, measurement of β-hCG is crucial to clarify pregnancy location and prognosis. Produced primarily by the syncytiotrophoblast in the placenta, β-hCG is detectable in the blood by the second week of pregnancy until a peak at 10–12 weeks [72]. A single measure of β-hCG is insufficient to clarify pregnancy prognosis, and serial β-hCG levels are commonly used to monitor early pregnancies. The most recent recommendations for β-hCG trends in early pregnancy, derived from a retrospective review of 1005 patients with PUL, suggest the minimum β-hCG rise of an IUP is 35 % in 2 days [73]. A β-hCG rise less than 35 % in 2 days has a positive predictive value of 96.2 %, a negative predictive value of 69.7 %, and an overall accuracy of 80.2 % in predicting EP. Conversely, in this study, in patients eventually diagnosed with miscarriages, the minimum expected β-hCG decline in 2 days is 36–47 % (depending on the starting β-hCG level). Β-hCG cut-offs, however, are not ironclad; using these cut-offs, 16.8 % of EPs and 7.7 % of IUPs would be misclassified solely using serial β-hCG levels. Obtaining a third β-hCG and early ultrasound decreased the misclassification of IUP to 2.7 % [73]. The expected rates of β-hCG rise and decline are the same for multiple pregnancies, following assisted reproduction, and in obese patients [74]. The absolute β-hCG values, however, may be higher in multiple pregnancies or lower in patients with elevated body mass index.

#### Serum progesterone

Serum progesterone has been explored as a possible serum marker for nonviable pregnancies, including EPs, as progesterone levels have been shown to be lower in ectopic and failing pregnancies than IUPs [75]. Several studies suggest a progesterone cut-off of 10 nanograms per milliliter (ng/mL) for the most accurate identification spontaneous EPs. In a meta-analysis including 4689 patients under 14 weeks gestational age with pain and/or bleeding, a serum progesterone level of less than 10 ng/mL predicted a non-viable pregnancy with a sensitivity of 66.5 % and specificity of 96.3 % [76]. The optimal cut-off may be higher in patients who received fertility treatments, as these patients often have multiple corpora lutea secreting progesterone and often receive exogenous progesterone. A cut-off of 30 ng/mL, 28–49 days after the last menstrual period, may be more appropriate in patients who received clomiphene citrate, while an optimal cut-off has yet to be identified in patients after IVF and is likely highly dependent on the number of days since embryo transfer [77, 78].

Serum progesterone levels, however, have been shown to misclassify more normal pregnancies than serial β-hCG measurements [79]. Serum progesterone also cannot further distinguish between miscarriages and EPs. While progesterone may highlight patients at greater risk for EP, it is insufficient in itself to discriminate between IUPs, miscarriages and EPs [80].

#### Other serum markers

Several studies have explored alternative serum markers of EP, focusing on proteins associated with placental, endometrial and/or corpus luteal functions, angiogenesis and inflammation [75]. These potential proteins include, but are not limited to: Inhibin A, which is produced by the corpus luteum; activin A, pregnancy-associated plasma protein-A (PAPP-A) and A Disintegrin and Metalloprotease-12 (ADAM-12) which are generated by the placenta; and vascular endothelial growth factor (VEGF), which, produced by a variety of cell types, is crucial for angiogenesis and may be upregulated in EP [75]. Various messenger and micro-RNA—regulators of downstream gene expression—may also be differentially expressed by an EP [81, 82].

Studies have also attempted to combine multiple measures; one such study incorporated VEGF, PAPP-A, and progesterone, and reported sensitivity of 97.7 % and specificity of 92.4 % in diagnosing EP, though this model has not been validated in further studies [83]. For many of these markers, studies are inconclusive, and for all markers, more research is needed before any of these supplants β-hCG as the primary serologic method of differentiating intra- and extrauterine pregnancies [81].

### Imaging

#### Discriminatory zone

Visualization of a gestational sac by transvaginal ultrasound (TVUS), confirming an intrauterine pregnancy (IUP), is expected at serum β-hCG levels above the “discriminatory zone.” The discriminatory zone was initially proposed as 6500 milli-international units (mIU)/mL in 1981, using transabdominal ultrasound [84]. With advances in ultrasound imaging, particularly with the use of transvaginal sonography, the discriminatory zone has been lowered to 1000 to 2000 mIU/mL [85].

Studies report that normal IUPs may still develop in patients with PULs and serum β-hCGs above the discriminatory zone. In a review of patients with PUL, nine women with β-hCGs above 2000 mIU/mL at the time of their TVUS developed intrauterine pregnancies; the highest β-hCG in this group was 4336 mIU/mL [86]. In patients with multiple pregnancies, the serum β-hCG at which an intrauterine gestational sac is seen can be higher than the discriminatory zone identified for singleton IUPs. One review reported a serum β-hCG of 9083 mIU/mL without definitive ultrasound findings in a patient later diagnosed with a triplet pregnancy [87]. Other factors such as obesity or uterine fibroids may also be associated with nonvisualization of an intrauterine gestational sac above the β-hCG discriminatory zone.

Serum β-hCG in the absence of definitive ultrasound findings should not be the sole factor in diagnosing pregnancy location or viability or dictating management, and that gestational age must be taken into account. A positive pregnancy test at any level in the absence of an intrauterine pregnancy should be approached as an EP until proven otherwise.

#### Accessory ultrasound findings

In the absence of definitive visualization of an EP, additional markers on ultrasound can increase a clinician’s suspicion for EP, and may be useful in conjunction with other clinical data. These include a thin endometrial stripe thickness and the presence of intraabdominal free fluid.

In a review of 591 patients with vaginal bleeding and PUL, IUPs had a significantly higher mean endometrial stripe thickness than miscarriages or EPs (17 mm versus 12 mm, respectively); no intrauterine pregnancies occurred in patients with endometrial stripes below 8 mm in thickness [88]. However, studies have not consistently shown a significant difference in endometrial stripe thickness between miscarriages and EPs [89]. Endometrial stripe thickness may indicate patients at higher risk for abnormal pregnancies but cannot reliably be used in isolation to diagnose EP [90].

A small amount of anechoic free fluid in the posterior cul de sac is normal in both intra- and extrauterine pregnancies [91]. Larger amounts of complex free fluid, particularly in Morrison’s pouch by the liver, may indicate rupture of an EP, and correlates well to hemoperitoneum observed intraoperatively [91, 92]. Hemoperitoneum can be assessed in the emergency setting using a Focused Assessment with Sonography for Trauma (FAST) scan, which is a bedside ultrasound assessing for free fluid in the perihepatic, perisplenic and pelvic space; the full trauma assessment, not applicable to patients with suspected EP, also includes the pericardial space [93]. The determination of extent of hemoperitoneum and need for intervention depends on clinician assessment and the patient's hemodynamic stability. Of note, if an ultrasound shows an IUP, the risk of EP is much reduced, though not zero. Though rare, the patient may still be at risk of heterotopic pregnancy, particularly following IVF [94].

#### Ultrasound diagnosis of tubal EP

**Fig. 1:**
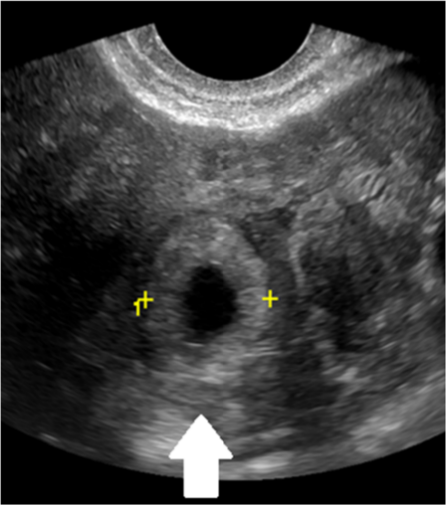
Tubal ectopic pregnancy by transvaginal ultrasound. The arrow indicates the ectopic gestation with a surrounding hyperechoic ring, called the ‘bagel’ or ‘tubal’ sign

**Fig. 2:**
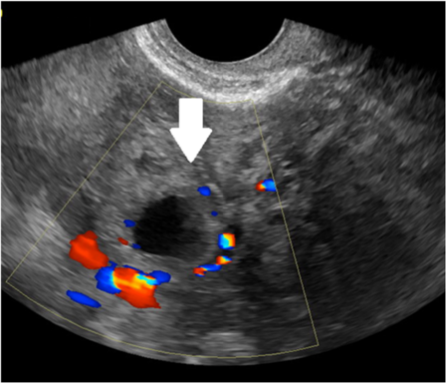
Tubal ectopic pregnancy by transvaginal ultrasound. The arrow indicates the ectopic gestation with circumferential Doppler flow, called the “Ring of Fire”

In women presenting with bleeding or pain, pregnancy location is often not definitively visualized on the initial ultrasound at presentation; however, diagnosis of EP by ultrasound is possible when following careful guidelines. Identification of a gestational sac and fetal pole, with or without cardiac activity, or a hyperechoic ring—called the ‘bagel’ or ‘tubal’ sign (Fig. 1)—with circumferential Doppler flow (Fig. 2) is highly suggestive of an ectopic pregnancy [95, 96]. If a suspicious mass moves separately from the ovary—called the ‘blob’ sign - the positive predictive value is above 90 % in a symptomatic woman with a positive serum b-hCG and no IUP on transvaginal ultrasound [97, 98].

#### Ultrasound findings specific to nontubal EPs

Ectopic pregnancies occurring in less common anatomic areas can also be identified by ultrasound according to specific criteria. Diagnostic criteria of each type of nontubal ectopic pregnancy are discussed below. All of these diagnostic criteria assume the absence of a visualized IUP.

**Fig. 3:**
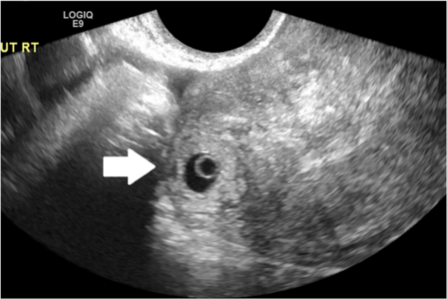
Interstitial ectopic pregnancy by transvaginal ultrasound. The arrow indicates thin (<5 mm) myometrium overlying the ectopic pregnancy. This finding by ultrasound, in combination with the lateral location of the gestation, has a reported specificity of 88-93 % but a sensitivity of just 40 % [101].

**Fig. 4:**
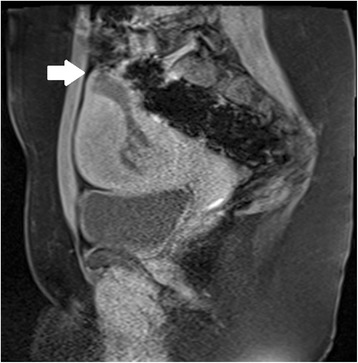
Interstitial ectopic pregnancy by magnetic resonance imaging, T1 weighted. The arrow indicates thin (<5 mm) myometrium overlying the ectopic pregnancy. In a stable patient, MRI may be useful in the confirmation of interstitial pregnancy location

**Fig. 5:**
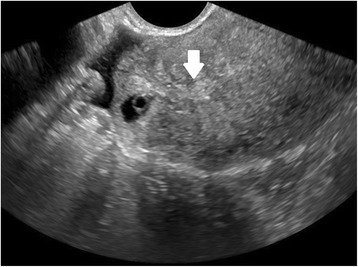
Interstitial ectopic pregnancy by transvaginal ultrasound. The arrow indicates the ‘interstitial line,’ extending from the endometrium to the cornua, abutting the suspicious mass

Ultrasound criteria for diagnosis of an interstitial ectopic pregnancy include a gestational sac at least 1 cm lateral to the edge of the uterine cavity, with a thin (5 mm or less) layer of overlying myometrium surrounding it (Figs. 3 and 4) [99, 100]. An ‘interstitial line’ may also be seen (Fig. 5) [101].

**Fig. 6:**
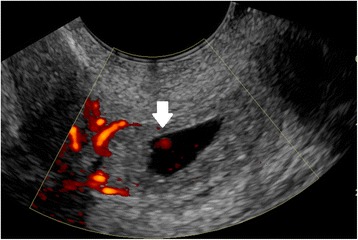
Cervical ectopic pregnancy by transvaginal ultrasound. Doppler shows circumferential flow. The arrow indicates Doppler flow inside the gestational sac, associated with the embryo. Such Doppler flow will not be found in a spontaneous abortion, which may slide down into a similar position at the cervix

A cervical EP is identified on ultrasound by a distended cervical canal containing a gestational sac with peripheral Doppler flow (Fig. 6), below a closed internal cervical os [102, 103]. The ‘sliding organ’ sign, or movement when pressure is applied with the transvaginal probe, is associated with spontaneous abortions in progress and should be absent in a cervical ectopic pregnancy.

**Fig. 7:**
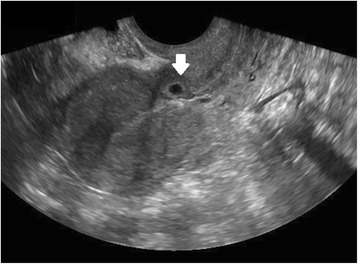
Cesarean scar ectopic pregnancy by transvaginal ultrasound. The arrow shows the gestational sac implanted in the region of the cesarean scar, clearly outside the endometrial canal

**Fig. 8:**
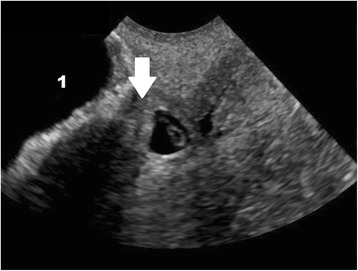
Cesarean scar ectopic pregnancy by transvaginal ultrasound. The arrow indicates the thin myometrium (3 mm) between the bladder (indicated with the number 1) and the gestational sac

Diagnostic criteria for a cesarean scar EP by ultrasound include visualization of the gestational sac at the site of the prior hysterotomy (Fig. 7), outside the endometrial cavity [104]. The myometrium should be very thin (1–3 mm) or absent between the gestational sac and the bladder (Fig. 8). A negative ‘sliding organ’ and the presence of peripheral Doppler flow are expected [9].

On ultrasound or MRI, intramural EPs should be completely surrounded by myometrium circumferentially, with no communication with the intrauterine cavity [69, 105]. Intramural EPs are notoriously challenging to diagnose on ultrasound and have been mistaken for fibroids or intrauterine pregnancies [106].

Ovarian EPs may be suspected by ultrasound when a hypoechogenic area is seen surrounded by a wide echogenic ring with peripheral Doppler flow. Some may be completely surrounded by ovarian cortex. [106, 107] A fetal pole is seldom present [107]. When pressure is applied with the transvaginal probe, an ovarian EP will move with the ovary, and should be connected to the uterus by the ovarian ligament [107, 108]. These EP can be difficult to differentiate from ovarian cysts, which may have a similar appearance and peripheral Doppler flow [108]. Given the difficulty of making this diagnosis by imaging, laparoscopy is often required for definitive diagnosis [107].

Abdominal EP is rare, and ultrasound guidelines are few. Suggested guidelines include visualization of an extrauterine gestational sac, fetus and/or placenta, with no myometrium seen between the fetus and urinary bladder [109]. This gestational sac or fetus will be in unusually close proximity to the anterior abdominal wall, and may be surrounded by loops of bowel. There should be no evidence of more common ectopic implantation sites, such as the fallopian tubes or cesarean section scar [110].

#### Endometrial sampling

At many medical centers, a patient with a PUL and an abnormal β-hCG trend as described above may receive methotrexate (MTX) without precise diagnosis [111]. Up to 40 % of these patients, however, may ultimately have failing IUPs, unnecessarily exposing these patients to MTX [112]. While not universally employed, endometrial sampling may allow these patients to avoid unnecessary treatment with MTX [113].

Identification of villi on endometrial sampling is diagnostic of a failed IUP, and in such cases, no further treatment is usually needed [5, 114, 115]. Serum β-hCG should also be checked the day after endometrial sampling; a decline of 15–20 % after sampling indicates the disruption of a failing IUP, even if no villi are identified [5]. A postoperative plateau or increase in the β-hCG value strongly suggests an EP. A patient with an adequate decrease in the β-hCG level after sampling can be monitored with serial β-hCG measurements until levels are undetectable, or until pathological evaluation of the curettage specimen shows chorionic villi [116].

The standard endometrial sampling method for PUL is D&C, though these are associated with greater cost and anesthesia requirement than outpatient procedures [112, 117, 118]. Endometrial biopsy pipelles, while effective in screening for endometrial carcinoma, have insufficient sensitivity and specificity to replace D&C in the diagnosis of PUL [119, 120]. Karman cannulas, attached to hand-held suction devices, are as efficacious as D&C for diagnosing endometrial pathology and are frequently used for the evacuation of first trimester pregnancies in the outpatient setting [121–124]. In a review of 45 patients with PUL and abnormal β-hCG trends after IVF, over two-thirds of patients were diagnosed with failing IUP by final pathology and/or falling β-hCG, and were spared MTX [116]. It is unknown whether this device performs similarly in spontaneous pregnancies.

### Medical management

The most common interventions for the treatment of EP are medical management with systemic MTX and surgical removal of the pregnancy. Medical management of EP with MTX has been demonstrated to be more cost-effective than surgical management while maintaining similar treatment success and future fertility [38, 125–127]. Injections of hyperosmolar glucose into tubal EPs have been studied, but have significantly higher failure rates than standard medical or surgical management and are not recommended [128–130].

MTX is a dihydrofolate reductase inhibitor, disrupting DNA and RNA precursor synthesis; it targets rapidly dividing cells and, in an EP, disrupts primarily trophoblastic tissue [131]. Its use as treatment for EP was first reported in 1982 [132]. The most common side effects associated with MTX treatment for EP include pelvic pain, nausea, headaches, abdominal pain, and dermatitis. Less common side effects include mucositis, diarrhea, and alopecia [127, 133].

 Several studies have demonstrated the efficacy of intramuscular (IM) MTX for treatment of EP, though success rates are inversely correlated to β-hCG levels [134]. In a meta-analysis including 503 women with EPs treated with single dose MTX, successful treatment, defined as avoidance of surgery, for initial β-hCG levels between 1000 and 1999 mIU/mL was 94.4 %, compared with just 81.8 % in patients with starting β-hCG levels of 10,000 to 150,000 mIU/mL [134]. A β-hCG above 5000 mIU/mL was proposed as a relative contraindication to treatment with MTX, with a success rate of just 85 %.

#### Pre-methotrexate assessment

Before treatment with MTX, blood work should be obtained to assess hematologic, hepatic and renal function; a chest x-ray should be considered in patients with active pulmonary disease. The patient’s Rhesus (Rh) status must also be obtained in order to determine the need for Rho(D) immune globulin therapy, required in Rh negative patients. A pelvic ultrasound should be obtained to characterize any ectopic mass and exclude a concomitant IUP. Several contraindications for treatment of EP with MTX exist (Table 1) [135]. Patients with ectopic pregnancies and relative contraindications to treatment with methotrexate may receive the medication if deemed appropriate by the clinician; these patients should be hemodynamically stable and well counseled and have capacity to make the decision [6].

**Table 1:** Absolute and relative contraindications of treatment of EP with MTX

Absolute Contraindications	Relative Contraindications
Clinical instability or significant pain suggestive of ruptured EP	Presence of fetal cardiac activity
Heterotopic pregnancy with viable and desired IUP	β-hCG level over 5000 mIU/mL
Liver function tests more than 2 times the upper limit of normal	An ectopic mass size greater than 4 cm in largest dimension
White blood cell count of <1500/uL	Patient refusal of blood transfusion
Platelet count <100,000/uL	Patient inability to follow up
Creatinine ≥1.5 mg/dL	
Current breastfeeding	
Active pulmonary disease	
Active peptic ulcer disease	
Moderate to severe anemia	
Sensitivity to methotrexate	

Patients should be advised to stop taking prenatal vitamins, as the folate supplementation will counteract the action of MTX. Patients should also avoid excessive sunlight due to possible MTX-induced dermatitis; nonsteroidal anti-inflammatory drugs, which may delay renal excretion of MTX; alcohol, which may lead to elevation of hepatic enzymes; and sexual activity, vigorous physical activity and pelvic exams, which could lead to rupture of the EP [136].

#### Systemic methotrexate

##### Single dose methotrexate

The single dose regimen consists of an IM injection of MTX (50 mg/m2 of body surface area), with administration of additional doses at weekly intervals in patients with an inadequate response (Table 2) [6]. Repeat injections are permitted every 7 days for up to 4 doses; a second dose is needed in 20 % or more of patients, while less than 1 % of patients require 3 or more doses [137]. The single dose regimen is associated with fewer side effects as compared to other regimens [6, 138, 139].

**Table 2:** Single dose methotrexate (MTX) for treatment of ectopic pregnancy

	Day 1	Day 4	Day 7
Labs	1. β-hCG	β-hCG	1. β-hCG
	2. Safety labs (complete blood count, BUN, creatinine, AST, ALT)		2. Safety labs
	3. Blood type and antibody screen		
Action	Give MTX (50 mg/m2 of body surface area IM)	no action	β-hCG decline <15 % from Day 4 to Day 7: MTX, return to day 1 of protocol. Repeat MTX up to a total of 4 doses
			β-hCG decline >15 %: Check β-hCG at 1 week intervals until zero.

#### Two dose methotrexate

A two dose regimen has been proposed by Barnhart and colleagues (Table 3) [133]. In their prospective study of 101 women with a mean serum β-hCG at treatment initiation of 2013 mIU/mL, the success rate, defined as avoidance of surgery, was 87 %. Patients suffered just mild and transient side effects, including nausea, headaches and abdominal pain, despite the lack of leucovorin supplementation.

**Table 3:** Two dose methotrexate (MTX) for treatment of ectopic pregnancy

	Day 0	Day 4	Day 7	Day 11	Day 14
Labs	1. β-hCG	β-hCG	1. β-hCG	β-hCG	1. β-hCG
	2. Safety labs (complete blood count, BUN, creatinine, AST, ALT)		2. Safety labs		2. Safety labs
	3. Blood type and antibody screen				
Action	Give MTX (50 mg/m2 of body surface area IM)	Give MTX (50 mg/m2 of body surface area IM)	β-hCG decline <15 % from Day 4 to Day 7: Give MTX	β-hCG decline <15 % from Day 7 to Day 11: Give MTX	β-hCG decline <15 % from Day 11 to Day 14: Refer for surgery
			β-hCG decline >15 % from Day 4 to Day 7: Check β-hCG at 1 week intervals until zero.	β-hCG decline >15 % from Day 7 to Day 11: Check β-hCG at 1 week intervals until zero.	β-hCG decline >15 % from Day 11 to Day 14: Check β-hCG at 1 week intervals until zero.

#### Multiple dose methotrexate

The multiple dose regimen was derived from chemotherapeutic regimens for gestational trophoblastic disease, involving administration of MTX and leucovorin (folinic acid) on alternating days for 8 days or until the β-hCG falls by 15 % from its peak value (Table 4) [6]. Up to 50 % of patients will not require the full 8 day regimen [6]. Leucovorin is administered to counteract the mechanism of MTX to limit side effects.

**Table 4:** Multiple dose methotrexate (MTX) and leucovorin (LEU) for treatment of ectopic pregnancy

	Day 1	Day 2	Day 3	Day 4	Day 5	Day 6	Day 7	Day 8
Labs	1. β-hCG		β-hCG		β-hCG		β-hCG	
	2. Safety labs (complete blood count, BUN, creatinine, AST, ALT)							
	3. Blood type and antibody screen							
Action	MTX (1 mg/kg, IM)	LEU (0.1 mg/kg, IM)	β-hCG decline <15 % from Day 1 to Day 3: Give MTX	LEU	β-hCG decline <15 % from Day 3 to Day 5: Give MTX	LEU	β-hCG decline <15 % from Day 5 to Day 7: Give MTX	LEU
			β-hCG decline >15 % from Day 1 to Day 3: Check β-hCG at one week intervals until zero.		β-hCG decline >15 % from Day 3 to Day 5: Check β-hCG at one week intervals until zero.		β-hCG decline >15 % from Day 5 to Day 7: Check β-hCG at one week intervals until zero.	

The reported success rates among the dosing regimens vary in the literature [127, 137, 138]. A recent randomized controlled trial of 120 women receiving single or multiple dose MTX reported no difference in success rates, though the time until β-hCG normalization was longer following the single dose regimen (22.3 vs. 18.3 days, respectively) [140]. Conversely, a meta-analysis of 1327 EPs reported that the rate of successful treatment with multiple dose MTX was significantly higher than with single dose MTX (92.7 vs. 88.1 %, respectively) [137]. Side effects, including nausea, vomiting and alopecia, were less common in the single dose treatment group. Of note, both treatment regimens were more likely to be successful in patients reporting side effects of the MTX.

Few comparisons have been published involving the two dose regimen. A retrospective comparison of 87 women receiving either single or two dose MTX regimens reported comparable success rates of 87 and 90 % at mean starting serum hCGs of 4801 and 4278 mIU/mL, respectively, and no difference in side effects [141]. In the literature, it is unclear which of these (single, two or multiple dose) regimens is used most commonly, though single and multiple dose regimens are discussed more often than the two dose regimen; MTX dosing is likely dependent on the provider and/or institution.

Regardless of which treatment regimen is chosen, if the β-hCG level does not decline adequately—after the multiple dose regimen, or 4 doses of MTX in single or two dose regimens—surgical management should be considered. A continued rise in serum β-hCG throughout the multiple dose regimen or after 2 doses of single dose MTX may indicate higher risk of rupture of a tubal EP [6, 142]. Finally, medical management should be abandoned in favor of surgical management if the patient presents with hemodynamic instability or other clinical parameters concerning for ruptured EP, such as pain. If a patient’s serum β-hCG declines adequately and she requires no further intervention, the β-hCG level should be monitored weekly to an undetectable level. On average, the β-hCG normalizes in 2 to 3 weeks, but can take up to 8 weeks in patients with higher starting β-hCG levels [6, 143].

### Surgical management

Surgical management is indicated in patients with contraindications to medical treatment as described in the previous section, hemodynamic compromise or other clinical signs of ruptured EP including pain or evidence of intra-abdominal bleeding, and according to patient preference.

The standard surgical intervention was laparotomy until the laparoscopic approach was introduced in 1973 by Shapiro and Adler; it has since gained wide acceptance [144]. Three prospective randomized trials have demonstrated the superiority of a laparoscopic approach over laparotomy in terms of lower blood loss, pain medication requirement, length of hospital stay and cost [145–148]. Reproductive outcomes, including rates of recurrent EP and subsequent IUP, are not significantly different between laparoscopy and laparotomy [149].

Regardless of the mode of abdominal entry, two methods of excision of a tubal EP have been extensively reported: Salpingectomy, or removal of the fallopian tube in part or in full, and salpingostomy (also called salpingotomy), or removal of the EP through a tubal incision while leaving the tube in situ. Salpingectomy is recommended in cases of extensive tubal damage and/or rupture, uncontrolled bleeding, prior tubal sterilization, or a large tubal EP (5 cm or more in diameter) [143]. The surgical approach is also determined by the status of the patient’s contralateral fallopian tube, the patient’s plans for future fertility, and surgeon comfort or preference.

#### Salpingectomy

Despite being termed “radical” in the literature, salpingectomy results in similar rates of subsequent IUP and ectopic recurrence as compared to salpingostomy. A randomized control trial of 446 women undergoing salpingostomy or salpingectomy reported similar recurrent EP and ongoing pregnancy rates between groups: 8 and 60.7 %, respectively, after salpingostomy and 5 and 56.2 %, respectively, after salpingectomy [39]. Persistent trophoblastic tissue, which usually requires treatment with MTX, was more common after salpingostomy (7 %) than after salpingectomy (<1 %). Of note, 43 women (20 %) randomized to salpingostomy underwent an intra-operative conversion to salpingectomy during the initial surgery due to uncontrolled bleeding. Similarly, in a review of 1064 women with EPs attempting subsequent conception, the rates of intrauterine pregnancies within 2 years were not significantly different, at 67 % after salpingectomy and 76 % after salpingostomy [38]. The rate of EP recurrence was also similar between groups, or 18.5 % overall. Following salpingectomy, if final pathologic analysis of the fallopian tube demonstrates evidence of a tubal gestation, no follow up β-hCG levels or any other assessment is needed.

#### Salpingostomy

Intraoperatively, if salpingostomy is planned, dilute vasopressin can be injected at the planned incision site for additional hemostasis [143]. After a 1–2 cm linear incision is made with electrocautery, laser or scissors over the bulging ectopic gestation, the contents are removed using forceps or high pressure irrigation, also called hydrodissection [143, 150–152]. The use of hydrodissection to flush out gestational products may be preferable to piecemeal removal with forceps, as the latter can lead to incomplete removal of trophoblastic tissue [4]. The tubal incision can be left open to heal by secondary intention or sutured closed; a Cochrane review reported an insignificant difference in rates of recurrent EP and subsequent IUP between the two techniques [148].

After salpingostomy, weekly β-hCG measurements are necessary to rule out persistent trophoblastic tissue, which can occur in up to 20 % of cases [153]. Administration of a single dose of intratubal MTX intraoperatively or IM MTX within 24 h postoperatively has been shown to decrease the rate of persistent trophoblastic tissue (from 14.5–17.5 to 0–1.9 %) [154, 155].

### Expectant management

A carefully selected subset of patients may be candidates for expectant management of tubal EP. Studies suggest that well-counseled, stable women with EPs and serum β-hCG of 175–200 mIU/mL and declining may be candidates for expectant management.

In an observational study of 107 patients diagnosed with tubal EP by transvaginal ultrasound (a mass separate from the ovary), expectant management was offered to asymptomatic patients without fetal cardiac activity [156]. Expectant management was discontinued due to severe pain or failure of the serum β-hCG to decline on sequential measurements. Ninety-six percent of women with a β-hCG of 175 mIU/mL or less did not require other treatment, compared to 66 % of those with β-hCG of 175–1500 mIU/mL and just 21 % of those with β-hCG above 1500 mIU/mL. Expectant management was more likely to be successful in patients with serum progesterone below 10 nmol/L (3.1 ng/mL), gestational age less than 6 weeks, and EP mass greater than 15 mm. Similarly, a prospective observational study of 118 patients with EPs managed expectantly reported that 88 % resolved with β-hCG levels below 200 mIU/mL, as opposed to just 25 % with β-hCG levels above 2000 mIU/mL [157].

The American College of Obstetricians and Gynecologists recommends that patients with EPs and serum β-hCG less than 200 mIU/mL and decreasing (though this is not strictly defined) are potential candidates for expectant management [136]. Patients undergoing expectant management for EP must be reliable for follow up, and willing and able to accept the risks of EP rupture, hemorrhage and emergency surgery.

### Management of nontubal EP

#### Ovarian EP

Management of ovarian EP is most commonly surgical, and little data is available on the medical management of this condition [6]. Successful treatment of ovarian EPs with systemic MTX alone has been described, using either the single or multiple dose regimens, up to a serum β-hCG of 5201 mIU/mL [158, 159]. Systemic MTX has also been described, following limited biopsy of a suspected ovarian EP, to address residual trophoblastic tissue [160, 161]. Successful management with transvaginal or laparoscopic injections of 50 mg of MTX directly into the ovarian EP, with β-hCG levels up to 12,075 mIU/mL, has also been reported [162, 163].

Often, laparoscopy is required for diagnosis, at which point definitive surgical management is often completed [107]. Management of ovarian EPs is primary surgical, and laparoscopic surgery has become the standard for management of hemodynamically stable patients with ovarian EPs [164, 165]. Resection of the EP and retention of the ovary is a reasonable surgical objective, particularly in patients desiring future fertility. This resection has most commonly taken the form of an ovarian wedge resection, attempting to remove as little normal ovarian tissue as possible [165]. In reports of surgical management of ovarian ectopic, hemostasis is obtained with electrocautery or ultrasonic energy; the latter is less damaging to the surrounding ovarian cortex [166, 167].

#### Cervical EP

Management of cervical pregnancies may be medical or surgical, with many centers utilizing a combination of approaches. The use of single or multiple dose systemic MTX and/or local MTX has been described in case reports and small series [168–170]. A series of 38 cervical EPs treated with local MTX, with additional local potassium chloride (KCl) in the presence of fetal cardiac activity, reported an overall success rate of 87 %; all failures had fetal cardiac activity [171]. In a review of 52 cervical EPs, 61.5 % were successfully treated with upfront systemic or local MTX [172]. Gestational age greater than 9 weeks, β-hCG titer over 10,000 mIU/mL, presence of fetal cardiac activity or fetal crown-rump length greater than 10 mm were associated with failure of upfront MTX.

Dilation and curettage is seldom used in isolation as a first line treatment, given the risk of hemorrhage; in a review of 15 cases with mean gestational age of 8.9 weeks, the risk of hysterectomy was 40 % [103]. Methods for decreasing the risk of bleeding include injection of vasoconstricting agents into the cervix, such as dilute vasopressin, or placement of cervical stay sutures [173]. Placement of intracervical catheter for tamponade, such as a 30 mL foley catheter, has also been described [174]. In the presence of fetal cardiac activity, preoperative injection of feticides may decrease the risk of hemorrhage [175].

Uterine artery embolization (UAE) may have a role in preventing or controlling hemorrhage; case series have reported both prophylactic UAE prior to medical and/or surgical management, or emergent use to control hemorrhage [170, 176–178]. This therapy is not currently recommended for women who wish to conceive in the future, as its ramifications for fertility have not been conclusively described.

#### Cesarean scar EP

Interruption of a cesarean scar EP upon diagnosis is recommended, given the risk of hemorrhage, hysterectomy and maternal morbidity [179, 180]. Live births resulting from a cesarean scar ectopic implantation have been described; however, these deliveries are frequently associated with hemorrhage and emergent cesarean hysterectomy [9, 181, 182]. In a series of 10 patients with cesarean scar EPs with fetal cardiac activity who elected for expectant management, 4 patients (40 %) had live births, 3 of whom (75 %) required hysterectomies; overall, 80 % required hysterectomies [183].

Medical management with single or multiple dose systemic MTX regimens has been described. Patients with serum β-hCG greater than 6000 mIU/mL may be at higher risk of requiring additional therapies, including local MTX, D&C or uterine artery embolization (UAE) [9, 64]. Local injections of MTX or KCl have also been described, usually in conjunction with systemic MTX or other surgical management (D&C or hysteroscopy) [184].

Several surgical approaches have also been reported, with the benefit of leading to more rapid resolution of β-hCG levels as compared to medical management [185]. Regardless of the chosen treatment modality, serum β-hCG should be followed to zero, as persistent trophoblastic tissue may occur after any medical or surgical treatments except hysterectomy [9, 186]. In patients undergoing upfront surgical management, D&C alone is often complicated by hemorrhage. In a meta-analysis of 21 cases, 76 % required further treatment, and 14 % required hysterectomy [64]. Initial steps for managing hemorrhage include tamponade with a transcervical catheter and hemostatic cervical cerclage sutures [187]. UAE has been used as both hemorrhage prophylaxis and salvage therapy in the event of hemorrhage [188]. UAE is not currently recommended for patients desiring future fertility.

Hysteroscopic resection of cesarean scar EPs has been performed successfully and without complication using biopolar or ‘electric’ loops, in patients with serum β-hCG up to 28,333 mIU/mL [186, 189, 190]. Hysteroscopic resection is not recommended when the residual myometrium is less than 3 mm, given the risk of anterior wall perforation and bladder injury [191, 192].

**Fig. 9:**
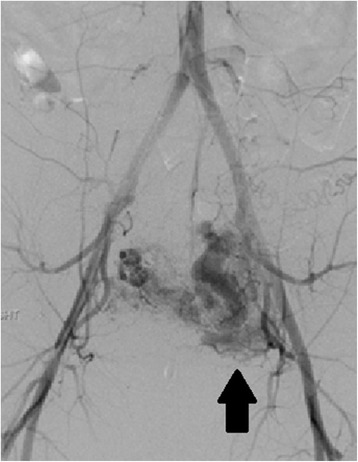
Left uterine artery arterio-venous malformation (AVM) by pelvic angiogram. This patient had undergone an uncomplicated ultrasound-guided D&C for a 10 week size cesarean scar ectopic pregnancy 2 months prior to presentation with vaginal bleeding and diagnosis of a left uterine artery AVM (arrow). The AVM was embolized with coils, but the patient required emergent hysterectomy for hemorrhage

Transabdominal excision of these lesions has been described by laparotomy, and standard or robotic-assisted laparoscopy [191]. Resection also allows for revision of the lower uterine segment, which theoretically may reduce risk for recurrence [193]. Laparotomy may be indicated in patients with suspected uterine rupture and hemodynamic instability, and hysterectomy may be required for otherwise uncontrollable hemorrhage [194]. Definitive management with total laparoscopic hysterectomy has also been described, in a patient with a starting β-hCG of 155,009 mIU/mL who failed treatment with local KCl and multiple dose MTX [195]. Of note, complications of medical or surgical management include formation of arterio-venous malformations, which are prone to bleeding; in one series of 60 cesarean scar EPs, this occurred at a rate of 8.5 %, requiring UAE or hysterectomy (Fig. 9) [183].

#### Interstitial EP

In patients who are hemodynamically stable without evidence of rupture of the interstitial EP, non-surgical management may be appropriate. Both single dose and multiple dose MTX regimens have been used to treat interstitial EPs with comparable success, ranging from 66 to 100 % [196]. UAE has also been successfully used as an adjunct to these therapies [197, 198]. Local MTX has also been used; a meta-analysis of 11 cases reported a success rate of 86 %, up to a serum hCG of 35,000 mIU/mL [4].

Surgical intervention is indicated following failed medical management, according to patient preference, or when the patient demonstrates hemodynamic instability and/or findings concerning for rupture of an interstitial EP, including pain or evidence of hemoperitoneum on imaging. Laparotomy and hysterectomy were formerly first line treatment, likely due to late diagnosis of interstitial pregnancies and higher rates of rupture and hemorrhage. These methods may still be necessary in patients with hemodynamic instability and severe hemorrhage.

Minimally invasive surgeries are increasingly pursued as imaging modalities allow for earlier diagnosis. Small case series have described ultrasound or laparoscopy-guided dilation and curettage [199–201]. Several laparoscopic surgical approaches have been described, including cornuostomy, salpingostomy, and cornual resection.

Cornuostomy entails injection of dilute vasopressin at the cornua followed by a linear incision, through which the gestation is removed with blunt and/or sharp dissection or hydrodissection, after which the incision is closed with absorbable suture [4]. Case series have also described successful surgical management with placement of an Endoloop around the base of the cornua before or after excision for both hemostasis and closure [202]. Less commonly, salpingostomy for interstitial ectopic has been reported, which is most appropriate for interstitial EP less than 3.5 cm, given the smaller incision with limited visualization [203, 204].

Cornual resection has been recommended for surgical management of more advanced interstitial pregnancies (greater than 3–4 cm) [12, 205]. This technique entails injection of dilute vasopressin followed by a circumferential incision using scissors or an energy source—electrosurgical or ultrasonic—preferably 1–2 cm above the cornual pregnancy to allow for redundant serosa and myometrium for closure [11, 12]. This incision should be closed in layers akin to a myomectomy closure. The fallopian tube adjacent to this cornua should also be excised. UAE has also been used as a prophylactic measure before laparoscopic cornual resection [206].

#### Intramural EP

As with other types and locations of EP, management of intramural EPs is largely dictated by patients’ clinical stability at presentation. In clinically stable patients with intramural EPs diagnosed by imaging, medical management is an option. In case reports, intramural EPs have been treated with single or multiple dose systemic MTX, successful in patients with serum β-hCG up to 25,140 mIU/ml [207–209]. Successful management with local MTX and KCl for an intramural EP with fetal cardiac activity and a β-hCG of 74,872 mIU/mL has also been reported, as well as UAE for an intramural EP with a β-hCG of 12,250 mIU/mL [210, 211].

Most cases of intramural EP reported in the literature have been managed surgically via laparotomy, sometimes requiring hysterectomy, as many patients present with rupture of the EP and hemorrhage [13]. Given the increasing ability of noninvasive imaging to diagnose intramural EPs and the advancement of minimally invasive surgery, more recent case reports have described laparoscopic excision of intramural ectopic gestations [212, 213]. A surgical approach should be determined by a patient’s clinical stability, desire for future fertility, and location of the ectopic gestation.

#### Abdominal EP

Intervention for resolution of an abdominal EP is recommended upon diagnosis, given the extremely high risk for maternal morbidity; the mortality risk associated with abdominal EPs is nearly 8 times the rate with tubal EPs [16]. Rare reports detail expectant management in order to attain a live birth. Expectant management of abdominal EPs may potentially be considered when the diagnosis is made after 20 weeks of gestation in a healthy patient who can be followed very closely through a tertiary care center. The fetus should have no congenital malformations, and the placenta should be implanted away from the upper abdomen. Delivery is recommended at 34 weeks, and the placenta is often left in place given the risk for hemorrhage [214, 215].

Most abdominal EPs reported in the literature have been managed surgically; the operative approach must be tailored to the patient’s clinical presentation and stability, and the location of the EP. Abdominal EPs have been approached by laparoscopy or laparotomy, with or without prophylactic embolization of the placental bed; more recent cases in the literature have been managed laparoscopically in hemodynamically stable patients [216–218]. Intraoperative blood transfusion is common; in a meta-analysis, the highest transfusion rate was associated with hepatic (46 %) and retroperitoneal (40 %) implantations, while abdominal wall implantations had the lowest transfusion rate (14 %) [16].

When abdominal EPs are removed surgically at any gestational age—though more commonly after 20 weeks of gestation—the placenta can be left in place to avoid hemorrhage [16]. Embolization of the remaining placenta and/or administration of systemic MTX or mifepristone have been employed to hasten resolution of these retained placentas [219, 220]. The most common complication of an intraabdominal retained placenta is infection [16].

As diagnostic modalities have advanced and these pregnancies are diagnosed earlier, case reports of medical management for abdominal EP have been published. Medical management with systemic MTX and/or local injections of MTX or KCl has been reported, though nearly half may require subsequent surgical management [16, 221–223]. Despite logistic regression, a meta-analysis failed to identify risk factors for failed medical management [16].

#### Heterotopic pregnancies

Treatment of a HP is tailored to the specific EP location, and the patient’s clinical presentation and stability [78]. Medical management of tubal HPs includes local injections of KCl or a hyperosmolar glucose solution, though over half of tubal HPs managed with local KCl may require subsequent salpingectomy [17, 224]. Treatment with systemic or local MTX, a known teratogen, is contraindicated in the presence of a viable IUP [225]. Surgical management has been described more frequently, as patients with tubal HPs present more often with rupture and hemodynamic compromise than those with tubal EPs [226]. Salpingectomy is preferable to salpingostomy as persistent trophoblastic tissue cannot be monitored in the setting of ongoing IUP [78]. Patients with HPs suffer spontaneous abortions at higher rates than intrauterine-only pregnancies (up to 30 %) [18].

#### Nontubal HP

For the management of interstitial HPs, expectant management, aspiration or injection of hyperosmolar glucose of the interstitial HP, and cornual resections have been reported, leading to live birth [227–229]. One patient attempting expectant management required a laparotomy for rupture of the interstitial EP [227].

Cesarean HPs have been successfully managed using local KCl and/or aspiration of the gestation, or excision by laparoscopy or hysteroscopy [230]. Hysteroscopy carries the theoretical risk of disrupting an IUP due to the high pressure infusion of fluid.

Cervical HPs addressed with expectant management, local KCl or hyperosmolar glucose injections, extraction with forceps, suction curettage or hysteroscopic resection, with or without subsequent foley tamponade, have resulted in live birth. Rare case reports also detail cerclage placement following intervention. Following this range of interventions, a review of 30 cases reported a live birth rate of 80 % [231].

Abdominal HPs are rarely encountered, though live birth after local injection of KCl into the abdominal pregnancy has been reported in 3 cases [232]. Ovarian HPs are similarly rare; live birth after local hyperosmolar glucose injection has been reported, as well as after laparoscopic wedge resection; surgical intervention carries the theoretical risk of interrupting hormonal support of the coexisting IUP by the corpus luteum [233, 234].

### Recurrence and future fertility

The risk of recurrence of tubal EP ranges from 5 to 25 % [38–40, 235]. The risk of recurrent EP is not affected by treatment modality—medical or surgical—or surgical procedure [38]. In a randomized controlled study of 446 women undergoing surgical management for tubal EP, the recurrence rate was similar after salpingostomy (8 %) and salpingectomy (5 %) [39].

A review of 53 cases of prior interstitial EP reported a recurrence rate of 9.4 % following either medical or surgical management [236]. In patients with a prior interstitial EP, data is limited regarding the risk of uterine rupture in a subsequent IUP, though uterine rupture has been reported after both expectant management and cornual resection [237, 238]. Vaginal deliveries have been reported following cornuostomy or cornual resection; the optimal mode of delivery in this group remains to be determined [12].

The reported rate of recurrent cesarean scar EP is highly variable, as high as 25 % in small series [239, 240]. Risk factors for recurrence are bulging of the prior cesarean scar EP into the uterovesical fold, initial presentation with irregular vaginal bleeding or pain, early termination (≤56 days) of the first cesarean scar EP, prior cesarean delivery at a rural community hospital and thin lower uterine segment (5 mm or less at the time of diagnosis of recurrent cesarean scar EP) [241].

The risk of recurrent cervical EP appears to be low: One recurrence was noted in a series of 34 pregnant women with prior cervical EP treated with several different modalities [67]. The data are insufficient to comment on subsequent IUP and recurrence rates in patients with prior ovarian, intramural or abdominal EPs. Rates of recurrence and IUP after HP have not been extensively reported in the literature, and likely depend on the location of the HP and the treatment modality.

Regardless of ectopic location, conception is not recommended for 3 months after exposure to MTX, though data for this recommendation is lacking [6]. Results of population-based studies of pregnancy outcomes after a prior tubal EP are encouraging, and independent of treatment modality. The rates of IUP have been shown to be similar following salpingectomy and salpingostomy in several large series [39, 40]. Additionally, among 1064 women with prior tubal EPs attempting conception, the rates of IUP within 2 years were similar among salpingectomy (67 %), salpingostomy (76 %), and medical management (76 %) [38]. After two prior EPs, however, the rate of subsequent IUP may be as low as 4 % [235].

## Conclusions

Ectopic pregnancy is a relatively common clinical scenario in general gynecology and reproductive medicine. While tubal pregnancies are the most common, EPs can occur throughout the abdomen and pelvis. Treatment in stable patients is often medical, though patients meeting certain clinical criteria or with EPs outside the fallopian tube may require differing and/or more invasive treatment, including excision by laparoscopy or, less commonly, laparotomy. Of patients with tubal EPs, the likelihood of future IUP is high and independent of treatment modality.

## Abbreviations

ADAM-12: A Disintegrin and Metalloprotease-12
ART: Assisted reproductive technology
D&C: Dilation and curettage
E2: Estradiol
EP: Ectopic pregnancy
FAST: Focused assessment with sonography for trauma
GnRH: Gonadotropin-releasing hormone
hCG: Human chorionic gonadotropin
HP: Heterotopic pregnancy
IM: Intramuscular
IUD: Intrauterine device
IUP: Intrauterine pregnancy
IVF: In vitro fertilization
KCl: Potassium chloride
LEU: Leucovorin
MTX: Methotrexate
NO: Nitric oxide
PAPPA: Pregnancy-associated plasma protein-A
PUL: Pregnancy of unknown location
TVUS: Transvaginal ultrasound
UAE: Uterine artery embolization
VEGF: Vascular endothelial growth factor
